# Extended Access to Hormonal Contraception in Pharmacies: A Survey among Swiss Pharmacists

**DOI:** 10.3390/pharmacy8040210

**Published:** 2020-11-10

**Authors:** Tamara Yous, Samuel Allemann, Monika Lutters

**Affiliations:** 1 Department of Medical Sciences, Private University of the Principality of Liechtenstein, 9495 Triesen, Liechtenstein; 2 pharmaSuisse, Swiss Pharmacists Association, 3097 Bern-Liebefeld, Switzerland; Samuel.Allemann@pharmasuisse.org; 3 Clinical Pharmacy, Cantonal Hospital of Baden, 5404 Baden, Switzerland; monika.lutters@ksb.ch

**Keywords:** hormonal contraceptives, birth control, community pharmacist, pharmaceutical services, pharmacies

## Abstract

Background: Worldwide the availability to Hormonal Contraceptives (HC) varies from over the counter (OTC) to prescription-only access. In various countries pharmacists are allowed to prescribe HC, although conditions may be different. In Switzerland, HC require a prescription from a physician, although Swiss law allows pharmacists to dispense prescription-only medications in justified exceptional cases without a valid physician’s prescription. This study aimed to identify current dispensing practices for HC in Swiss pharmacies, pharmacists’ knowledge about HC, and their opinion and interest about expanding access to HC. Methods: Web-based survey among Swiss pharmacists. Results: This survey was completed by 397 registered pharmacists and 331 (83%) were included for analysis. The survey showed that 21% of respondents regularly dispense HC without prescription and that a high number of participants are either very interested (57%, *n* = 189) or rather interested (33%, *n* = 110) in extended pharmacy access to HC. The majority did not or rather not support physician’s prescription-only status (77%, *n* = 256) or OTC availability (94%, *n* = 310). Furthermore, surveyed pharmacists are willing to train for contraception services (90%, *n* = 299). According to participants, resistance of physicians is the most relevant barrier to this service (88%, *n* = 292). Conclusion: Surveyed pharmacists are interested in extended access to HC.

## 1. Introduction

Enovid^®^, the first combined oral contraceptive, was approved by the FDA in 1960 [1]. During the last 60 years, progress has been made in terms of user friendliness, effectiveness, and safety of Hormonal Contraceptives (HC). The benefit–risk ratio of HC has been continuously improved and advantages outweigh disadvantages for most patients [2]. Serious complications, such as venous thromboembolism or brain and myocardial infarction, are rare in women of reproductive age [3,4]. Nevertheless, theoretical safety concerns and outdated guidelines still restrict best practice and timely access to effective contraception [2,5].

A global analysis regarding the access to HC showed that only 31% of 147 countries studied implemented prescription-only models [6]. In several countries HC are already available in pharmacies, although conditions may vary from over the counter (OTC) availability to pharmacist prescribing. While in the United States of America (US) prescription status remains unchanged, many states allow prescribing of HC by pharmacists [6,7,8]. Furthermore, New Zealand has reclassified selected HC to allow supply by pharmacists, although first time users and women aged less than 16 years are ineligible [9,10]. Expanding access to HC is currently also being evaluated in several European countries, e.g., Belgium, Germany, and the United Kingdom [11,12,13,14]. For example, Parsons et al. showed that trained community pharmacists provided appropriate contraception service and that the pharmacy is a reasonable site to provide HC [12]. It has been demonstrated that relevant contraindications can be identified with existing tools (e.g., a checklist) and that trained pharmacists successfully identified women without contraindications to HC [3,15,16]. In contrast, Mobark et al. found suboptimal counselling practice in the United Arab Emirates and the authors hypothesized that pharmacists are probably inadequately trained for providing this service [17].

Gynecological examination is not considered necessary for prescription of HC, but a careful review of medical history as well as blood pressure measurement are recommended [3,15,18,19,20]. Surveys from the US have repeatedly pointed out women’s interest in pharmacy access to HC and it has been shown that providers generally support extended access [21,22,23]. HC have been assessed as sufficiently safe by the ACOG (American College of Obstetricians and Gynecologists) and ACCP (American College of Clinical Pharmacy) and they advise to release these drugs from physician’s prescription-only [24,25].

Community pharmacies have recently extended their role in primary health care and pharmacists have demonstrated their potential to improve population’s health outcome [26]. Besides advising patients on their medications, pharmacists provide numerous preventive health services such as immunization or screening for sexually transmitted infections [26,27,28,29,30].

In Switzerland, pharmacy training consists of a 3-year Bachelor and a 2-year Master degree. Since 2018, registered pharmacists are required to complete a specialist degree to work independently in community pharmacy. Swiss pharmacists already play an important role in reproductive health as they offer counselling on Emergency Contraceptives (EC) [31,32]. In Switzerland, EC are available since 2002 from pharmacists without the need of a physician’s prescription. However, HC still requires a prescription from a physician. Although Swiss law allows pharmacists to dispense prescription-only drugs (including HC) in justified exceptional cases without a valid physician’s prescription, initiation of HC or changing between methods is currently not supported [32]. With hormonal contraception service from pharmacists and the extended access to HC, e.g., initiation of HC, renewing of prescriptions, or changes between different products could be authorized. Giving the availability of EC without prescription, together with the recommendations for HC and the extension of the role of pharmacists, the question rises whether the access to HC in Switzerland should be extended to pharmacies without physician’s prescription. This would be possible under a new law to simplify access to prescription-only medications in pharmacies [32]. However, no data is available regarding pharmacists’ interest in extended access to birth control in Switzerland. We conducted this study to determine pharmacists’ opinion regarding expanding pharmacy access to HC. Furthermore, we aimed to identify current dispensing practices as well as potential barriers for women to access HC. Moreover, pharmacists were interrogated about their knowledge in order to identify potential knowledge gaps about HC as well as potential issues for the implementation of contraception service in pharmacies.

## 2. Materials and Methods

We conducted a web-based survey among Swiss pharmacists in 2020 (23 April to 01 June 2020). Recruitment occurred through the printed Swiss Pharmacists’ Journal (pharmaJournal) and a monthly electronic newsletter (pharma30), both published by the Swiss Association of Pharmacists (pharmaSuisse, Bern-Liebefeld, Switzerland) and distributed to approximately 6000 registered pharmacists [33].

The questionnaire was administered using the web-based survey tool SoSci Survey (Version 3.2.05-i) [34]. Furthermore, it was specifically designed for Swiss Pharmacists in German and French. Some parts of the questionnaire are based on previous publications and were adopted to the Swiss health care system [7,11,35]. As proposed by Kallus, the quality of the translation was verified by back translation [36]. The survey was tested in two pilot trials. It covered demographics and a total of 19 questions with predetermined answer choices. The questionnaire included the following topics: current supply practice of HC and EC, potential barriers to access HC, opinion on extended access and contraceptive knowledge, motivation to provide hormonal contraception service, willingness for training and potential issues for the implementation of extended access to HC. At the end, a comment field was provided.

Although pharmacist prescribing is not a common expression in Switzerland, we used the term “pharmacist prescribing” in the context of extended access to HC. For our survey we defined that hormonal contraception service and extended access to HC would include counselling and screening for contraindications as well as prescribing and dispensing HC. In case of contraindications a referral to a physician was foreseen.

The Cantonal Ethics Committee of Zurich confirmed that the authorization from the ethics committee is not required for this study.

Analysis was conducted using SPSS (IBM Corp. Released 2016. IBM SPSS Statistics for Windows, Version 24.0. Armonk, NY, USA) and Microsoft^®^ Office Excel (Version 16.35). Only fully completed questionnaires were included in the evaluation but robustness of data was tested with the full data set. Data was analyzed using descriptive statistics. For the hypothesis testing, groups were formed according to the hypothesis and grouped where appropriate. Test analysis was performed using chi-square, Fisher, Kruskal Wallis, or Mann-Whitney-U Test as well as bivariate correlation analysis (spearman’s correlation). In case of significant differences, post hoc tests were performed, and Bonferroni method was used to adjust significance levels.

## 3. Results

### 3.1. Participants

A total of 397 pharmacists participated in this study, which corresponds to around 7% of pharmacists receiving the printed and electronic communications with the link to the survey. A total of 331 questionnaires were included in the analysis (83% of responses). Participants took on average 12 min (SD: 4 min; min–max: 5–30 min) to complete the questionnaire. Study population’s characteristics are summarized in Table 1. Pharmacists worked mainly in community pharmacies and participated from all geographical regions of Switzerland (data not shown).

### 3.2. Current Practice

A majority of participants dispense HC without prescription either in exceptional situations (75%, *n* = 248) or regularly (21%, *n* = 70). Most pharmacists dispense HC upon prescription on a daily basis (Table 2). Nearly 40% dispense HC without prescription at least once a week. Only a small number of pharmacists never dispense HC without prescription.

Male pharmacists more regularly dispense HC without prescription compared to female pharmacists (*p* = 0.003). Participants who fill more HC prescriptions also more frequently dispense HC without prescription (r = 0.165) and pharmacists frequently counselling on EC more often dispense HC without prescription (r = 0.327).

Participants were requested to choose the three most common situations out of a list of five, in which they provide HC without prescription. Pharmacists seem to dispense without prescription mostly because of expired prescriptions (93%, *n* = 308) or non-accessibility of physicians (67%, *n* = 221). The following situations were selected equally frequently: women from abroad demand for HC (49%, *n* = 161) or the prescription is filed in another pharmacy (48%, *n* = 158). Lost prescription was the least frequent choice (32%, *n* = 106).

### 3.3. Pharmacists’ Interest in Providing Hormonal Contraception Service

Overall, 90% of participants were either very interested (57%, *n* = 189) or rather interested (33%, *n* = 110) to provide hormonal contraception service. Nearly all participating pharmacists (90%, *n* = 299) were willing to train for this service. A minority was willing to train for more than 3 days (14%, *n* = 46). Pharmacists considered this service as important (60%, *n* = 200) or rather important (27%, *n* = 90) for public health. The majority (89%, *n* = 295) answered that this service should be further evaluated. No differences in interest regarding sex, age, working area (urban vs. countryside), or language (German vs. French) could be observed (*p* > 0.05).

### 3.4. Potential Barriers to Access Hormonal Contraception

**Figure 1:**
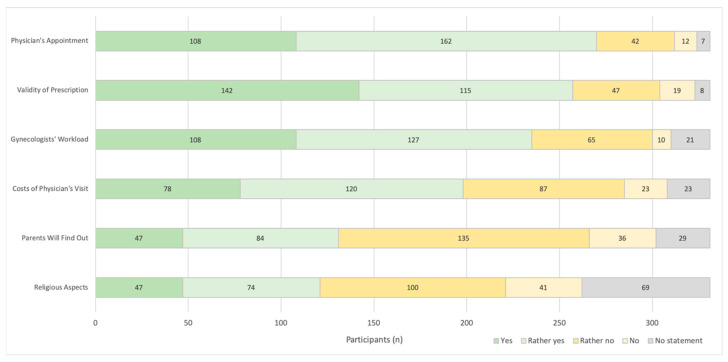
Potential Barriers. “What do you think are possible barriers to access Hormonal Contraceptives from the concerned women’s point of view?” (*n* = 331).

When asking about potential barriers to access HC from the concerned women’s point of view, the majority (82%, *n* = 270) answered that the necessity for a physician’s appointment can (rather) limit the access (Figure 1). Furthermore, validity period of prescriptions and workload of gynecologists seemed to be other important barriers.

Statistical analysis showed that pharmacists working in urban areas (*p* = 0.047; data not shown) or younger pharmacists (40 ± 11 vs. 43 ± 12 years (mean ± SD); *p* = 0.006) answered more frequently that costs for physician’s visit can be a barrier. Furthermore, younger pharmacists were more likely to report the fear that parents will find out (39 ± 11 vs. 43 ± 12 years (mean ± SD); *p* = 0.008) and religious aspects (39 ± 11 vs. 44 ± 12 years (mean ± SD); *p* = 0.003) can display barriers to access HC.

### 3.5. Extended Access to Hormonal Contraception

#### 3.5.1. Different Models and Inter-Professional Collaboration

According to participants, HC should not (77%, *n* = 256) or rather not (16%, *n* = 54) be available OTC. Furthermore, access solely upon physician’s prescription is regarded as not (35%, *n* = 116) or rather not (42%, *n* = 140) appropriate. This leads to a majority (rather) preferring models involving both physicians and pharmacists (76%, *n* = 252), but the requirement for an initial physician’s prescription was favored (91%, *n* = 300). Switching between different contraception methods is also considered a possible practice for pharmacists (67%, *n* = 222). Furthermore, participants were highly interested in inter-professional collaborations (78%, *n* = 258). Pharmacists from urban areas were significantly more interested than their colleagues from the countryside (*p* < 0.001; data not shown).

#### 3.5.2. Advantages of Pharmacy Access

**Figure 2:**
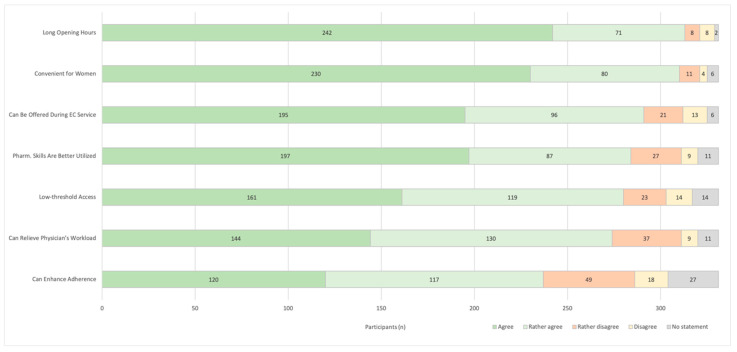
Advantages of Pharmacy Access. “What are the advantages of extended pharmacy access to Hormonal Contraceptives in your opinion?” (*n* = 331; EC = Emergency Contraceptive; Pharm = Pharmaceutical).

When asking about the advantages of pharmacy access for HC, most participants selected long opening hours as the main benefit (Figure 2). Moreover, this service is considered convenient for women and can be offered during counselling on EC. Participants working in the countryside significantly more often answered that this service is comfortable for women (*p* < 0.001; data not shown) and more younger pharmacists believed that this service can relieve physician’s workload (40 ± 12 vs. 45 ± 12 years (mean ± SD); *p* = 0.018).

#### 3.5.3. Pharmacist’s Concerns

**Figure 3:**
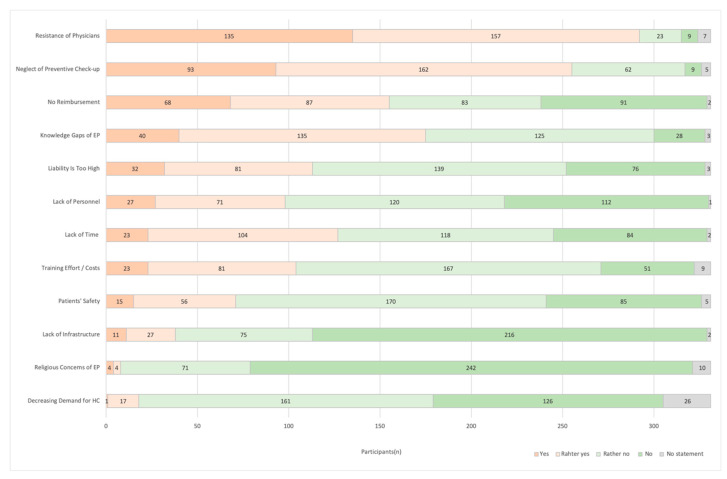
Pharmacist’s Concerns. “What are the current issues regarding the extended access to Hormonal Contraceptives in your opinion?” (*n* = 331; EP = Employees; HC = Hormonal Contraceptives).

Participants were mostly concerned about physicians’ resistance or the possible neglect of gynecological check-ups (Figure 3). The majority of participants answered that patients’ safety is rather not an issue and liability is not deemed too high.

#### 3.5.4. Patients’ Safety

**Figure 4:**
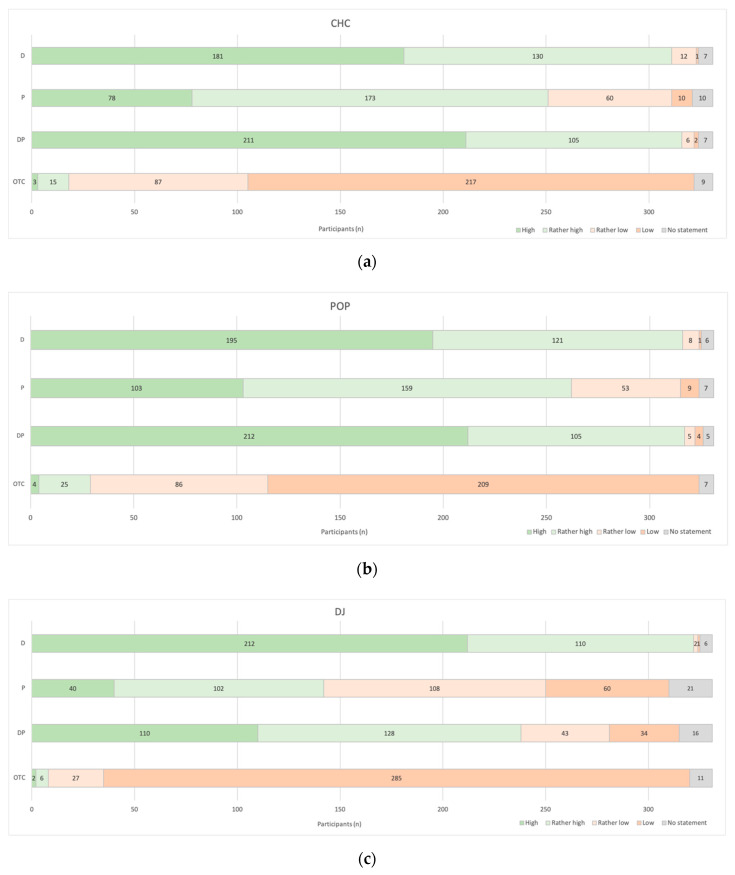
Patients’ Safety. “How do you assess patients’ safety for the different prescription models for CHC (**a**), POP (**b**), and DJ (**c**)?” (*n* = 331; CHC = Combined hormonal contraceptives; POP = Progesterone-only pills; DJ = Depot-Injection; D = Physician’s (doctor’s) prescription; P = Pharmacist’s prescription; DP = Initial physician’s prescription and follow up by pharmacists; OTC = Over the counter).

The opinion about safety was investigated separately for combined hormonal contraceptives (CHC), progesterone-only pills (POP) and depot injections (DJ) and is summarized in Figure 4. Pharmacist’s prescription for CHC and POP was rated more secure than for DJ. Combined prescription models were thought to be slightly safer than with physician’s prescription only. In general, OTC access models were considered less safe.

### 3.6. Knowledge and Skills

Pharmacists were asked to indicate their knowledge and skills for certain topics regarding contraception service. Participants mostly declared to have optimal or rather optimal knowledge about measuring blood pressure (89%, *n* = 293 or 10%, *n* = 33) and managing missed doses of HC (55%, *n* = 181 or 37%, *n* = 123). However, a high number of pharmacists declared rather deficient or deficient knowledge on selecting the best suitable contraception method (47%, *n* = 156 or 11%, *n* = 37). Furthermore, many pharmacists reported rather deficient or deficient knowledge on administering DJ (19%, *n* = 63 or 46%, *n* = 152). Compared with males, female participants indicated significantly better knowledge about preventive examinations (*p* < 0.001; data not shown) and management of missed doses (*p* < 0.001; data not shown).

Half of the participants declared to feel partially prepared for pharmacy prescription of CHC (*n* = 165) and POP (*n* = 164) by their pharmaceutical studies. A total of 31% (*n* = 101) felt already well enough trained to prescribe CHC and 30% (*n* = 98) for POP. Overall, younger pharmacists felt better prepared by their pharmaceutical studies to prescribe CHC (37 ± 9 vs. 45 ± 12 years (mean ± SD); *p* < 0.001) and POP (37 ± 10 vs. 45 ± 12 years (mean ± SD); *p* < 0.001) than their older colleagues. Most pharmacists did not feel prepared to prescribe and administer DJ (57%, *n* = 189).

## 4. Discussion

### 4.1. Interpretation

This was the first survey assessing Swiss pharmacists’ opinion regarding extended access to HC and we found a high proportion of participants interested in providing hormonal contraception service. Unique conditions of the Swiss healthcare system (e.g., physicians dispensing drugs directly in their medical practices) and recent changes in the role of pharmacists (e.g., providing vaccination service) could contribute to interest in new services. Previous surveys among pharmacists and also among women from other countries showed that they were generally supportive of extended access to HC [11,21,22,23,35,37,38,39,40,41]. So far, it is not known whether this service will find agreement among women in Switzerland.

Another finding of this survey is that more than 20% of participants are regularly challenged with situations where no valid prescription is available for HC. Even though in Switzerland, prescriptions for HC are normally issued for a certain number of packages and are valid for one to two years, the frequent situations in which pharmacists already dispense HC without a valid prescription could further explain their interest in this service. Nieuwinckel et al. found Flemish pharmacists to be in a similar situation and concluded that “*this practice anticipates what many health care professionals already suggested or could agree with: extending a prescription to the pharmacist*” [11].

Interestingly, our survey revealed that participants who dispense HC more frequently also dispense more HC without prescription. This fact could be explained as pharmacists who dispense more prescribed HC probably also have more experience with HC and, therefore, might be more willing to take the responsibility to dispense HC without prescription. Additionally, participants with frequent counselling on EC more often dispense HC without prescription, maybe trying to prevent the need for EC with this practice.

Pharmacist’s profession is currently undergoing a change. Our survey showed that participants are motivated to complete additional training in order to provide hormonal contraception service which is in agreement with previous research [11]. Although participants generally already reported to be knowledgeable about certain aspects, we also identified some knowledge gaps in this survey, e.g., selecting contraception methods or administration of depot injections. As far as we can tell, both topics are currently not addressed in the pharmacy curriculum at Swiss universities. Interestingly, younger pharmacists felt better prepared by their pharmaceutical studies. A possible explanation could be the recategorization of EC some years ago and the implementation of this topic into the curriculum at universities. With this change, younger pharmacists probably not only gained more knowledge about EC, but also about reproductive methods in general.

Authorizing pharmacists to prescribe HC would be a major change in the health care system and we revealed some concerns. Resistance from physicians was seen as most relevant. The majority of surveyed pharmacists support inter-professional collaboration (e.g., regular case discussions and cooperation with physicians), which could lead to new opportunities and new working models. Pharmacists from urban areas were more likely to be interested in inter-professional models. Urban areas offer more possibilities for such collaborations and probably there is less concern about competing interests. In contrast, participants from the countryside were more likely to answer that hormonal contraception service from pharmacies is comfortable for women and the reduced availability of physicians/gynecologists in rural areas might explain this finding. Moreover, enriched pharmacists’ knowledge could be beneficial for women seeking counselling, might relieve physician’s workload and allow them to focus on services which only they can provide [13]. Participants were concerned that women could neglect gynecological check-ups, which was also reported in a survey among US pharmacists [35]. Preventive check-ups are very important interventions for cancer screening, but according to the literature they do not contribute substantially to safe and effective use of HC and are unrelated to the prescription of HC [5,18]. A study from the US showed that women not using HC and, therefore, without a need for a physician’s prescription, still regularly obtain preventive care [22]. To address this concern, pharmacists providing contraception services should encourage women for regular gynecological check-ups.

In general, patients’ safety was not of relevant concern among our participants. However, the survey clearly revealed that pharmacists are worried about patients’ safety if HC were available OTC. A recently published study from Germany reported “conservative attitude” among pharmacists regarding a possible switch of HC to OTC availability [14]. According to the medical association ACOG, requiring a pharmacist to access oral contraceptives only replaces one barrier with another [24]. Giving the current practice in Switzerland, drug availability from pharmacists is generally not seen as a barrier and is a common approach in Switzerland. Pharmacists prescribing has various advantages that have also been addressed in this study. In contrast to OTC availability, pharmacists prescribing HC would ensure patient-pharmacist-interaction. In our survey, the majority of participants rated patients’ safety slightly higher with physician’s prescription, but we detected relevant knowledge gaps possibly contributing to this cautious perception. Additional training and providing specific screening tools could address safety concerns and support pharmacists. In contrast to research from the US, liability was not a main concern in our survey [35].

The importance of reimbursement of the service was found to be controversial. Swiss basic health insurances only cover preventive gynecological check-up every three years. HC are not reimbursed by basic health insurance, unless women have a supplementary insurance offering coverage. Interestingly, more younger pharmacists indicated that costs for a physician’s visit can display a barrier to access HC. Maybe they expect the service to be less expensive in pharmacies because no other examinations are performed. Reimbursement is probably not equally important to all women and other advantages such as long opening hours or easily and timely accessible service could be considered more valuable. Extended access to HC would leave the choice to women on where to obtain HC.

### 4.2. Strengths and Limitations

A limitation of this study is that it was conducted during a work intensive period for pharmacists because of the Covid-19 pandemic, probably contributing to the low participation. Consequently, the results may not accurately reflect opinions from all pharmacists in Switzerland. Men and French-speaking pharmacists were slightly under-represented compared to the whole member base of pharmaSuisse. Furthermore, participants received no incentives, which could have led to an over-representation of pharmacists with a high interest in the topic. Our study has also various strengths. For the first time, we provide information about opinion and interest of Swiss community pharmacists for extended access to HC. Moreover, the questionnaire is based on previous research and has been specifically adjusted to the Swiss health care system [7,28,29]. The questionnaire was provided in two languages, both French and German, using state-of-the-art translation methodology [30]. Furthermore, all language regions and age groups are covered. Noteworthy, our study was not financially supported by interest groups.

### 4.3. Open Question and Further Research

Besides the perspective of pharmacists, further research should investigate the opinions from women using HC and other health care providers in Switzerland.

### 4.4. Relevance of these Findings

Our study shows a possibility to extend access to HC in Switzerland with a potential service in Swiss pharmacies, which has already been successfully implemented in other countries. Furthermore, this service would support the strategy of the Swiss government, to simplify access to medications and to extend the role of pharmacists. Nevertheless, having pharmacists as direct providers of contraception care is a major shift in responsibility and this needs to be addressed together with all stakeholders. This study can inform policy makers and stakeholders about pharmacists’ opinion, possible concerns, and potential hurdles.

## 5. Conclusions

Swiss pharmacists who participated in this study are interested and motivated to provide contraception service. They are willing to train for this service and extend the role of pharmacists.

## Figures and Tables

**Table 1:** Participant Characteristics.

**Age (years)**	***n*** **(%)**
<30	64 (19%)
30–39	115 (35%)
40–49	65 (20%)
50–59	62 (19%)
≥60	25 (7%)
Average Age (SD)	41 (12)
Median (min–max)	38 (24–77)
**Gender**	***n*** **(%)**
Female	257 (78%)
Male	74 (22%)
**Work Setting**	***n*** **(%)**
Community Pharmacy	304 (92%)
Hospital Pharmacy	6 (2%)
Industry/Authority	7 (2%)
Others (e.g., University)	14 (4%)
**Pharmacy Location**	***n*** **(%)**
Countryside	94 (28%)
Urban	237 (72%)

*n* = 331.

**Table 2:** Current Practice. "How often do you dispense Hormonal Contraceptives (HC) or Emergency Contraceptives (EC) on average?”.

	≥1 Per Day	≥1 Per Week	≥1 Per Month	Never
HC with Rx	271 (82%)	53 (16%)	7 (2%)	0 (0%)
HC without Rx	29 (9%)	130 (39%)	161 (49%)	11 (3%)
EC	16 (5%)	141 (43%)	174 (52%)	0 (0%)

*n* = 331; EC = Emergency Contraceptives; HC = Hormonal Contraceptives; Rx = Prescription.
